# A “V”-Shaped Intraoral Lipoma on the Floor of the Mouth: A Case Report

**DOI:** 10.7759/cureus.30260

**Published:** 2022-10-13

**Authors:** Karthik Rajaram Mohan, Saramma Mathew Fenn, Ravikumar Pethagounder Thangavelu, Pallavi Vyapaka

**Affiliations:** 1 Department of Oral Medicine and Radiology, Vinayaka Mission's Sankarachariyar Dental College, Vinayaka Mission's Research Foundation, Salem, IND; 2 Conservative Dentistry and Endodontics, Vinayaka Mission's Sankarachariyar Dental College, Vinayaka Mission's Research Foundation, Salem, IND

**Keywords:** 940 nm diode laser, lipolysis, surgical excision, floor of the mouth, lipomas

## Abstract

A benign soft tissue tumor of mature fat cells is called a lipoma (adipocytes). Lipoma can develop anywhere on the body, although it is uncommon in the mouth. Lipomas that are superficially positioned are often yellowish in color, painless, soft, and non-fluctuating with a thin epithelial surface. As a result, a delicate pattern of blood vessels is frequently seen on the surface. Deeper lesions might not exhibit this finding and, as a result, are not as clinically recognized. Since the patients do not report any subjective clinical symptoms, the dentist often diagnoses such lipomas by accident. Deep-seated lipomas require specialist imaging procedures, such as contrast-enhanced computed tomography, magnetic resonance imaging, or ultrasound to determine their extent. Lipomas can range in size from tiny to large to enormous. Large lipomas typically feature a "slip sign" and a nodular surface. Giant-sized lipomas can have a diameter of up to 10 cm. Lipomas can be single or multicellular. Dercum's disease, Proteus syndrome, neurofibromatosis, and familial adenomatosis polyposis all exhibit lipomas in various locations. The preferred course of treatment for these oral lipomas is surgical removal. Such lipomas do not recur again. Lipoma comes in a number of tiny varieties. The traditional description is of a well-defined tumor made up of lobules of uniformly sized and shaped mature fat cells. The term "fibrolipoma" refers to lipomas that contain a sizable amount of fibrous connective tissue, "angiolipoma" refers to lipomas that contain numerous tiny blood vessels, "myxolipoma" refers to lipomas with a background of myxoid cells, and "spindle cell lipoma" refers to lipomas that contain a mixture of uniform spindle cells. When compared to a pleomorphic liposarcoma, the pleomorphic lipoma exhibits spindle cells and strange, hyperchromatic large cells, making it challenging for the pathologist to tell them apart. An intramuscular lipoma is a lipoma that invades skeletal muscle bundles. Because they are harder to entirely eradicate, intramuscular lipomas are more likely to reoccur.

## Introduction

A lipoma is a benign tumor of mature fat cells (adipocytes). The most common type of lipoma is a typical subcutaneous lipoma. It is regarded as a "universal tumor" since it can develop everywhere in the body, but it most commonly affects the back, shoulder, and neck. Subcutaneous lipoma is the most common; lipoma can also occur in other locations as well such as the intra-arterial, subsynovial, subdural, subfascial, parosteal, subserosal, submucosal (gastrointestinal tract), or extradural spaces (spine). Signs and symptoms of lipomas depend largely on the location and size of the lipomas. Patients may present with respiratory distress due to bronchial obstruction if either endobronchial or parenchymal lipoma occurs. Cardiac lipomas are located mainly subendocardially, are rarely found intramurally, and are normally unencapsulated; they appear as a yellow mass projecting into the cardiac chamber lesions. Patients with esophageal lipomas can present with obstruction, dysphagia, regurgitation, vomiting, and reflux; esophageal lipomas can be associated with aspiration and consecutive respiratory infections. Multiple lipomas occur in Dercum’s disease, gastrointestinal polyposis syndrome, Proteus syndrome, and neurofibromatosis. Tumors appear on the neck, back, and upper trunk in benign symmetrical lipomatosis (multiple symmetrical lipomatosis/disease/Launois-Bensaude Madelung's syndromes), with middle-aged men being more susceptible. There are primarily three types of lipoma: encapsulated, diffuse, and multiple. Encapsulated lipomas have a core of fatty tissue enclosed by a capsule. When diffuse lipomatosis first appears before age two, mature adipose tissue masses infiltrate large portions of a limb or the trunk. Diffuse lipomatosis is associated with tuberous sclerosis. Encephalocraniocutaneous lipomatosis includes cranial and ocular abnormalities and subcutaneous lipomas on the scalp with overlaying alopecia. The name "nevus psiloliparus" refers to the cutaneous component. Another manifestation of this hamartomatous illness is infiltrating lipoma of the face. Intermuscular lipomas and intramuscular lipomas have both termed the same name. There are several forms of invading lipoma. A clinically swollen and soft scalp results from a thickening of the subcutaneous fat of the scalp, known as lipedematous scalp. It might appear clinically as alopecia [1].

## Case presentation

**Figure 1:**
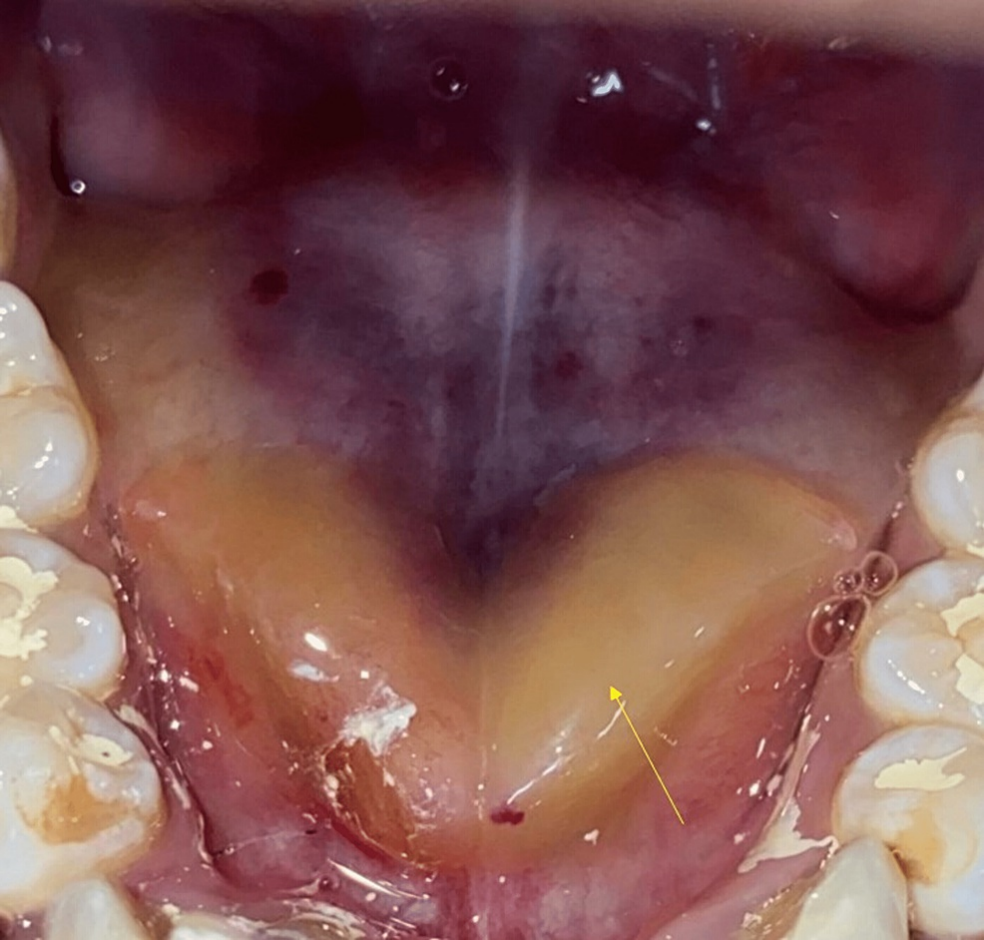
Intraoral examination revealed a yellowish colored swelling on the floor of the mouth resembling the shape of letter "V"

A 21-year-old female presented to our department for a routine dental checkup. Her medical history was non-contributory. Her personal history revealed that she had no harmful oral habits such as smoking or chewing tobacco. A general examination revealed that her vitals were stable. Extraoral examination did not reveal any cervical lymphadenopathy. Intraoral examination revealed an intraoral swelling on the floor of the mouth. On inspection, the swelling was yellowish in color and extending in the shape of the alphabet "V" on the floor of the mouth (Figure 1).

**Figure 2:**
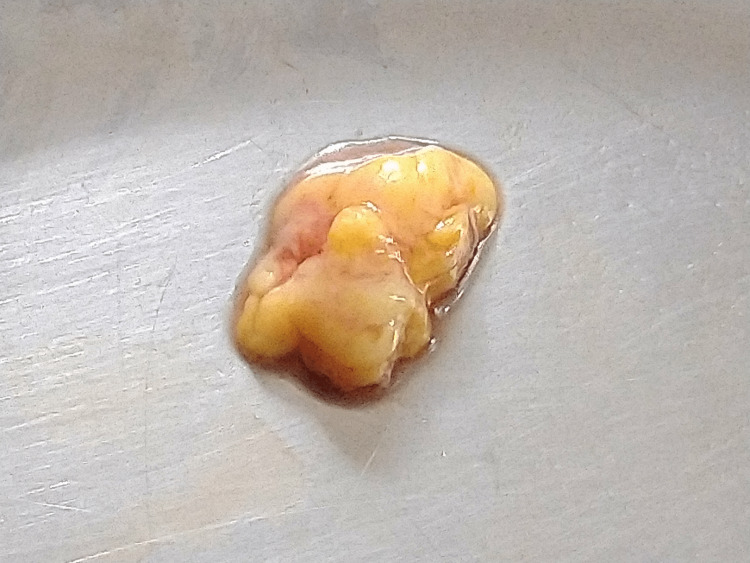
Excised specimen

On palpation, it was soft in consistency, non-tender, non-fluctuant, and non-reducible. The provisional diagnosis of intraoral lipoma was made. The clinical differential diagnosis considered were ranula, epidermoid cysts, and mucoepidermoid carcinoma of the floor of the mouth. Ranula usually has a superficial bluish tinge. Lipomas are generally yellow if located superficially because of the yellow-colored fat tissue. Entrapped ectodermal tissue from first and second branchial arches during its development in the third and fourth weeks of gestation is the usual cause of epidermoid cysts, which typically occur in the midline near the floor of the mouth. Dermoid cysts appear on the floor of the mouth and can lead to elevation of the tongue. Low-grade mucoepidermoid carcinoma of minor salivary glands also appears on the floor of the mouth. Mucoepidermoid carcinoma also has an increased tendency to develop cystic degeneration and clinically resembles mucocele without causing pain or discomfort [2]. Under local anesthesia, the swelling was excised and sent for histopathological evaluation (Figure 2).

**Figure 3:**
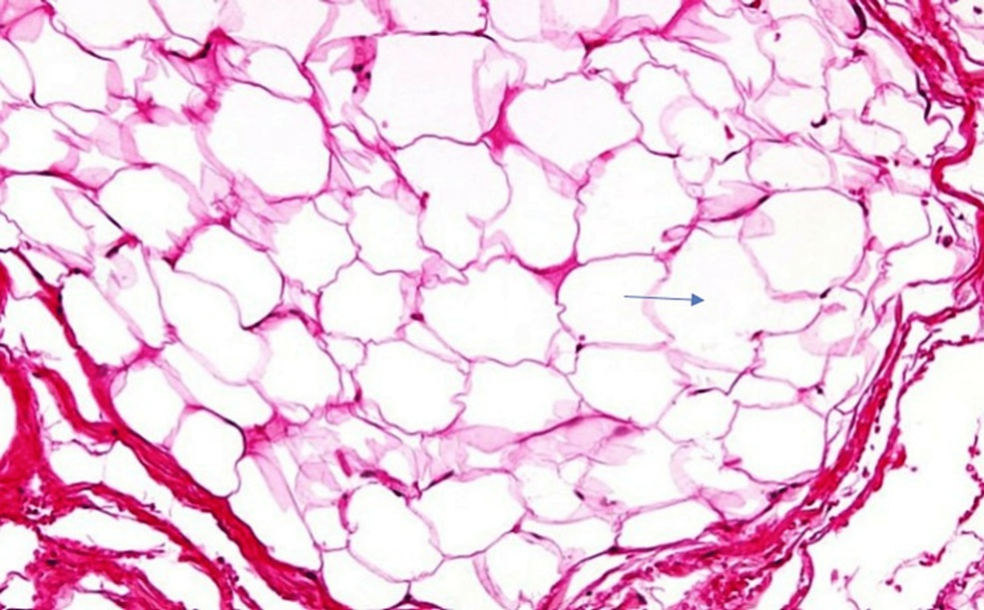
Histopathological photomicrograph (30x) showing numerous fat cells (adipocytes) with clear cytoplasm

The histopathological photomicrograph (30x) revealed adipocytes (fat cells) with clear cytoplasm (Figure 3). The patient did not turn for further clinical and radiological evaluation follow-up.

## Discussion

Etymology of lipoma

The word "lipoma" is derived from the Greek word "Liparein", which means "to persist, persevere". "Lipos" means fat. The word "Leip" is from the proto-Indo-European word meaning "to stick" and "to adhere." Lipoma is a benign tumor made of fat. Lipomas can occur at any age and frequently appear clinically as soft, slowly expanding swelling with no pain. Lipomas usually occur as solitary but also can appear as multiple lipomas. For example, numerous multiple lipomas occur in Dercum's disease, in which tender lipomas occur in the body involving the trunk. Multiple lipomas occur near the midline along the corpus callosum of the brain in Pai syndrome. Lipomas are tender due to the compression of nerve elements within such lipomas (adiposis dolorosa).

Incidence and prevalence

Lipoma occurs in the head and neck region in around 15% of cases and in the oral cavity in around 4.4% [1]. Furlong et al. stated that among a total of 125 lipoma cases (91 males, 33 females, and one unknown gender), around 5 ( 0.04%) (four classic lipomas and one spindle cell lipomas) cases occurred on the floor of the mouth [2]. Egido-Moreno et al. stated that lipoma occurs in about 7.4% (seven out of 95 cases, 56 women and 39 men) on the floor of the mouth [3].

Etiopathogenesis of lipomas

The etiopathology of Lipomas remains controversial. However, some suggested that hereditary chromosomal aberrations 12q 14.3 translocations, biochemical alterations such as hypercholesterolemia, and constant trauma predispose to lipomas [4].

Theories on lipoma

Hypertrophy Theory

The "hypertrophy theory" contends that these oral lesions may develop due to obesity and unintentional adipose tissue expansion. This theory less satisfactorily explains lesions that develop in regions devoid of preexisting adipose tissue. Unlike normal fatty tissue, they do not deplete by general metabolism during times of hunger [5].

Metaplasia Theory

Connective tissue cells virtually anywhere in the body can change to fat cells; the "metaplasia theory" contends that the genesis of lipomatous tissue results from the aberrant differentiation of in situ mesenchymal cells into lipoblasts [5]. The various clinical and histopathological classifications of lipomas are described in Table 1.

**Table 1:** Clinical and the histological types of lipomas

Clinical types of lipomas	Location
Subcutaneous	Flank, shoulder
Subsynovial, intra-articular	Elbow joint, knee joint
Intermuscular	Thigh, shoulder region
Parosteal	Under periosteum of bone
Subfascial	Limb, sole, palm
Subserosal	Retroperitoneum
Submucous	The gastrointestinal tract, Larynx
Extradural or epidural lipomas	spine
Esophageal lipomas	Esophagus
Laryngeal lipomas	Larynx
Cardio lipomas	Intra-atrial and ventricular chambers of the heart, commonly the right atrium and left ventricle
Intraglandular	Pancreas, Breast
Multiple lipomas	Trunk
The histological type of lipomas	Location
Intramuscular lipomas or myolipomas	Retroperitoneum, abdominal cavity
Osteolipomas	Bone
Neurofibrolipomas	Extremities
Osteochondrolipomas	Ischium of the pelvis
Spindle cell lipomas	Shoulder, posterior neck
Angiolipomas	Trunk, neck, upper arm, legs
Myxolipomas	Heart, tongue, oral cavity, epiglottis
Angiomyxolipoma	Subcutaneous tissues of the scalp
Sialolipomas or salivary gland lipomas	Parotid gland
Liposarcomas	Muscles of the limbs, abdomen

The most popular variety is subcutaneous encapsulated lipoma. It comprises overactive fat cells in normal fat that are grouped in lobules, divided by fibrous septa, and encased in a fragile capsule. Although it can happen anywhere on the body, it is more frequently observed in the neck, the back, the area around the shoulder, and the upper limbs. The skin that lies on top is typically of a normal color. The skin may be stretched over the tumor with dilated veins only in cases of very big lipomas [6].

The various literature reviews on cases of lipomas reported on the floor of the mouth are described in Table 2.

**Table 2:** Review of case reports of lipoma on the floor of the mouth

Author	Year	Age	Gender	Location	Conclusion
Coimbra et al. [6]	2006	29-year-old	Female	The floor of the mouth	Recurrence of lipomas is rare
Aguiar de Freitas et al. [7]	2009	29-91 years, mean age = 54.6 years	Female: high predilection	26 cases of lipomas; N=3 floor of the mouth	The benign nature of lipomas is the absence of its recurrence after surgical excision
Manor et al. [8]	2011	Mean age = 59.7 years	29 males; 29 females	N=6 floor of the mouth	Oral lipoma is rare. Lipomas are yellowish. They are benign, slow-growing, soft tissue neoplasm of adipocytes.
Raj et al. [9]	2014	72-year-old	Male	The floor of the mouth	Lipomas were considered in the differential diagnosis of painless, slow-growing swelling on the floor of the mouth.
Kumar et al. [10]	2014	72-year-old	Male	The lower left mental region	Intraoral lipomas are rare and noticed only during routine dental examinations. Most of them rarely cause pain, resulting in delays in seeking treatment. The patient’s concerns may be regarding aesthetics or discomfort.
Naruse et al. [11]	2015	Mean age = 59 years	11 males; 13 females	The floor of the mouth n=2	Lipoma is a benign tumor made of fat
Raghunath and Manjunatha [12]	2015	20-year-old Female	Female	The floor of the mouth	Lipomas are benign
Jeyaraj and Segha [13]	2017	37-year-old	Male	The floor of the mouth	Lipoma caused discomfort while eating, speaking, drinking, and swallowing
Gibson et al. [14]	2021	60-year-old	Male	The floor of the mouth	Lipoma on the floor of the mouth is painless
Sarfi et al. [15]	2021	64-year-old	Female	The floor of the mouth	Lipoma is an asymptomatic benign soft‐tissue neoplasm. The lesion is typical of its slow-growing nature, encapsulated. Surgical excision is the elective treatment in cases where the lesion is encapsulated and is easily separated from surrounding tissues. The relapse of this variant is uncommon, but long‐term follow‐up is mandatory.

Management of lipomas 

Stebbins et al. advocated the use of a 980-nm diode laser after lipolysis for successful removal of sizeable subcutaneous lipoma [16].The use of a syringe or a multi-injector device such as a mesorelle and mesogun are some of the injection strategies that have been described. Small, soft regions of localized fat, lipomas, post-liposuction deformities, skin contour irregularities brought on by traumatic fat necrosis, cellulite, post-fat grafting deformities, and depressed scar with adjacent areas of projecting fat are all indications for injection lipolysis. This approach should not be used to treat more extensive fat deposits (>500 ml), fat pads that are more than 3 cm thick, fibrous fat, or thinner fat deposits that are dispersed across a large surface area [17].

Complications of lipoma

When a lipoma is present for an extended period of time, it may change in some aspects. This is exceptionally accurate when there is a lipoma in the subcutaneous tissue of the thigh, buttock, or backward-facing lipoma. These alterations include (i) saponification, (ii) myxomatous degeneration (iii) calcification, (iv) infection, (v) repetitive trauma-induced ulceration, and (vi) malignant change (liposarcoma).

## Conclusions

Lipomas can occur anywhere in the body, called universal tumors, but intraoral lipomas are rare. Such intraoral lipomas are incidentally discovered during routine dental checkups. Intraoral lipomas are usually painless, soft in consistency, do not exhibit characteristic slip signs, and do not cause discomfort to the patient. Lipomas occur in solitary or multiple lesions. Lipomas can be small or large or can reach a giant size of 10cm, called giant lipomas. The signs and symptoms depend on their anatomical location in the human body. Lipomas in the larynx can cause stridor, and those in the esophagus can cause dysphagia. Lipomas that occur in the duodenum can cause intussusception or obstruction. Deep-seated lipomas are delineated only by magnetic resonance imaging (MRI). A careful clinical examination aid in the clinical diagnosis of lipomas and prevents unwanted radiographic procedures such as contrast-enhanced computed tomography, which are hazardous because of the ionizing radiation and anaphylactic reactions to contrast medium in some individuals.
